# Indolent Small Intestinal CD4+ T-cell Lymphoma Is a Distinct Entity with Unique Biologic and Clinical Features

**DOI:** 10.1371/journal.pone.0068343

**Published:** 2013-07-04

**Authors:** Elizabeth Margolskee, Vaidehi Jobanputra, Suzanne K. Lewis, Bachir Alobeid, Peter H. R. Green, Govind Bhagat

**Affiliations:** 1 Department of Pathology and Cell Biology, Columbia University Medical Center, New York, New York, United States of America; 2 Department of Medicine, Celiac Disease Center, Columbia University Medical Center, New York, New York, United States of America; UTHealth Medical School, United States of America

## Abstract

Enteropathy-associated T-cell lymphomas (EATL) are rare and generally aggressive types of peripheral T-cell lymphomas. Rare cases of primary, small intestinal CD4+ T-cell lymphomas with indolent behavior have been described, but are not well characterized. We describe morphologic, phenotypic, genomic and clinical features of 3 cases of indolent primary small intestinal CD4+ T-cell lymphomas. All patients presented with diarrhea and weight loss and were diagnosed with celiac disease refractory to a gluten free diet at referring institutions. Small intestinal biopsies showed crypt hyperplasia, villous atrophy and a dense lamina propria infiltrate of small-sized CD4+ T-cells often with CD7 downregulation or loss. Gastric and colonic involvement was also detected (n = 2 each). Persistent, clonal TCRβ gene rearrangement products were detected at multiple sites. SNP array analysis showed relative genomic stability, early in disease course, and non-recurrent genetic abnormalities, but complex changes were seen at disease transformation (n = 1). Two patients are alive with persistent disease (4.6 and 2.5 years post-diagnosis), despite immunomodulatory therapy; one died due to bowel perforation related to large cell transformation 11 years post-diagnosis. Unique pathobiologic features warrant designation of indolent small intestinal CD4+ T-cell lymphoma as a distinct entity, greater awareness of which would avoid misdiagnosis as EATL or an inflammatory disorder, especially celiac disease.

## Introduction

The gastrointestinal (GI) tract is the most common extranodal site for the occurrence or presentation of lymphomas, the majority of which are of B-cell origin. [1], [2] Peripheral T-cell lymphomas (PTCLs) account for approximately 15% of primary intestinal lymphomas. [3], [4] Secondary involvement of the GI tract by different subtypes of T- and NK-lineage lymphomas can be seen in up to 46% of cases at autopsy. [5] Enteropathy associated T-cell lymphoma (EATL) types I and II and extranodal NK/T-cell lymphoma, nasal type, are the most frequent types of lymphomas presenting with intestinal involvement.[6]–[8] Rarely, other types of PTCL such as ALK+ anaplastic large cell lymphoma and gamma-delta T-cell lymphoma can also arise in the GI tract or involve it secondarily.[9]–[11].

Primary T/NK-cell lymphomas of the intestine are associated with a poor prognosis and a high risk of bowel perforation.[6]–[8] However, rare cases of primary GI indolent lymphoproliferative disorders of CD8+ and CD4+ T-cell lineages have been described, mostly as sporadic case reports.[12]–[18] Recently, distinct phenotypic, biological and clinical features of indolent NK-cell lymphoproliferations of the GI tract were delineated in a series of cases. [19] Although morphologic and clinical features of indolent lymphomas of the T-cell lineage have been described, data regarding their immunophenotypic profiles and associated genomic abnormalities are limited.

Hence, we evaluated the pathologic, genomic and clinical characteristics of three cases of indolent CD4+ T cell lymphomas, primarily involving the small intestine. All displayed similar morphologic, immunophenotypic and clinical features and manifested non recurrent genetic abnormalities, distinct from other types of primary enteric T-cell lymphomas. [20] In conjunction with prior reports,[14]–[17] our findings suggest the existence of a unique and rare subtype of PTCL not recognized in the current WHO classification, which warrants greater awareness for correct diagnosis and optimal management. [20].

## Materials and Methods

### Case Selection

We searched our departmental database for cases of primary intestinal T-cell lymphomas diagnosed at our institution over 17 years (1996 and 2012) to identify cases that manifested features distinct from known types of PTCL. Laboratory test results were obtained from our laboratory information system and information regarding clinical presentation, imaging, serologic testing, therapy and follow-up were obtained from the treating physicians. All patients provided written informed consent for use of tissue samples for research, as well as inclusion in the clinical database of the Celiac Disease Center of Columbia University, in accordance with the regulations of the Columbia University Human Research Protection Program and protocols approved by the Institutional Review Board of Columbia University, New York, USA.

### Morphology, Immunohistochemistry and in situ Hybridization

Hematoxylin and eosin stained slides were reviewed for cyto-morphologic evaluation. A comprehensive immunohistochemical (IHC) staining panel was performed in all cases. Primary antibodies included CD3, CD5, CD8, CD20 and CD30 (DAKO, Carpinteria, CA, USA); CD2, CD7, CD25 and CD56, (Vector, Burlingame, CA, USA); CD4 (BioGenex, San Ramon, CA, USA); TCRγ (ThermoFisher, Waltham, MA, USA); perforin, Bcl6 and CD10 (Novocastra, Newcastle Upon Tyne, UK); granzyme-B (Chemicon, Temecula, CA, USA); T-cell intracellular antigen-1 (TIA-1) (Beckman Coulter, Fullerton, CA, USA); Ki-67 and ALK1 (Ventana, Tucson, AZ, USA); FoxP3 and SIRT1 (Abcam, Cambridge, MA, USA); and PD-1 (Cell Marque, Rocklin, CA, USA). IHC staining was performed with an automated staining machine (Universal Staining System, DAKO) after moist heat induced antigen retrieval and Envision Plus (DAKO) was used for visualization with Diaminobenzidine as the chromogen, according to standard protocols. In situ hybridization for EBV encoded small RNAs (EBER) was performed as per the manufacturer’s recommendations (Ventana, Tucson, AZ, USA).

### Flow Cytometry

Four color flow cytometric analysis was performed on cell suspensions from the tissue biopsies and peripheral blood samples (FACScan; Becton Dickinson, San Diego, CA, USA) using Cell Quest software (Becton Dickinson) according to standard procedures. The antigens evaluated included CD2, CD3, CD3ε, CD4, CD5, CD7, CD8, CD10, CD13, CD14, CD16/56, CD19, CD20, CD30, CD33, CD34, CD45, CD103, TCRβ, TCRγ and HLA-DR.

### T-cell Receptor Gene Rearrangement Analysis

Polymerase chain reaction to detect T-cell receptor (TCR)β gene rearrangement was performed on DNA extracted from fresh or formalin-fixed biopsies from stomach, duodenum, jejunum, and colon, as well as peripheral blood mononuclear cells using the ‘Biomed-2’ primers (InVivoScribe Technologies, San Diego, CA, USA), as described previously. [21].

### Single Nucleotide Polymorphism (SNP) Array Analysis

Copy number analysis was performed using the Affymetrix Genome-Wide Human SNP Array 6.0 or CytoScan HD (Affymetrix, Santa Clara, Calif., USA) on DNA extracted from tumor samples (Case 1, n = 1; Case 2, n = 3; and Case 3, n = 1, Tables 1 and 2) and from normal peripheral blood mononuclear cells (Case 3). Sample preparation, hybridization, and scanning were performed according to the manufacturer’s specifications. Analysis was performed using the Affymetrix Chromosome Analysis Suite 1.0 (ChAS). A `normal reference set´ of DNA from 50 samples, comprising 25 female and 25 male samples from individuals with no phenotypic abnormalities and normal karyotypes, run on the Affymetrix SNP Array 6.0 in our laboratory was also used for comparison with tumor samples. The reference model file provided by Affymetrix was used for CytoScan HD analysis.

**Table 1:** Clinical characteristics at presentation.

Case	Age/Sex	HLA-DQ typing	Presenting signs/symptoms (other abnormalities)	Duration of symptoms prior to diagnosis (years)	Endoscopic findings	Ann Arbor Stage at presentation	ECOG	IPI	Therapy	Outcome
1	53M	DQA1*05+ (half of the DQ2 heterodimer)	Diarrhea, weight loss, night sweats (deficient in vitamins A,E, D, K and Mg)	15	Mucosal nodularity, scalloping, erythema	IEB	1	Low risk (1)	budesonide	Alive with persistent lymphoma (4.6 years after diagnosis). Diarrhea well controlled. Requires vitamin supplementation
2	37M	DQ8+	Diarrhea, weight loss (deficient in vitamins D, K, and Iron)	3	Mucosal nodularity, scalloping	IE	0	Low risk (0)	budesonide, azathioprine, prednisone	Died due to sepsis after small bowel perforation (11 years after diagnosis)
3	50F	DQ2−DQ8-	Diarrhea, weight loss	2	Mucosal nodularity, scalloping	IE	1	Low risk (1)	budesonide	Alive with persistent lymphoma and microscopic colitis (2.5 years after diagnosis). Diarrhea well controlled

**Table 2:** Morphologic, immunophenotypic, and cytogenetic characteristics.

Case	Sites of involvement	Small intestine histopathology	Immunophenotype	Stomach (TCR)	Small intestine (TCR)	Colon (TCR)	Blood (TCR)	SNP array	Cancer associated genes
1	Duodenum, jejunum, ileum	TVA, small lymphocytes in LP	CD2+, CD3+, CD5+/−, CD7−, CD4+, CD8−, CD103−, TCRαβ+	ND	Clonal	PC	PC	No changes	None
2a (1998)	Duodenum, ileum, stomach, colon	TVA, small lymphocytes in LP	CD2+, CD3+, CD5+/−, CD7+/−, CD4+, CD8−, CD103−, TCRαβ+	Clonal	Clonal	Clonal	ND	19q13.31 loss	None
2b (2005)	Duodenum, ileum, stomach, colon	TVA, small lymphocytes in LP	CD2+, CD3+, CD5+/−, CD7+/−, CD4+, CD8−, CD103−, TCRαβ+	Clonal	Clonal	Clonal	Clonal	1p32.1 gain; 8q24.22 gain	*JUN, NDRG1, WISP1*
2c (2009)	Small intestine, liver, peripheral blood	TVA, small lymphocytes in LP, large lymphocytes in mucosa and submucosa, multifocal necrosis	CD2+, CD3+, CD5−, CD7−, CD4+, CD8−, CD25+, CD30+, Mum1/IRF4+, granzyme-B+, perforin+	Clonal	Clonal	Clonal	Clonal	1p36.12q21 gain; 15q21.2 gain; 17q21.2q31 gain; Xp22.11 gain; 7q11.22q23 LOH	*PAX7, SDHB, PDRM2, STAT3, PRDX4, ZFX, ELN*
3	Duodenum, terminal ileum, appendix, stomach, colon	Patchy STVA, small lymphocytes in LP	CD2+, CD3+, CD5+, CD7+, CD4+, CD8−, CD103−, TCRαβ+	Clonal	Clonal	Clonal	PC	Monosomy X	*ATRX, BCOR, ELF4, GATA1, GPC3, KDM5C, KDM6A, MED12, MLLT7, MSN, MTCP1, NONO, PHF6, SEPT6, SSX1, SSX2, SSX4, TFE3, WAS, WTX, ZRSR2, CRLF2, P2RY8*

TCR: T-cell receptor β gene rearrangement; TVA: total villous atrophy; STVA: subtotal villous atrophy; SNP: single nucleotide polymorphism; LOH: loss of heterozygosity; LP: lamina propria; ND: not done; PC: polyclonal.

## Results

### Clinical Characteristics

**Figure 1:**
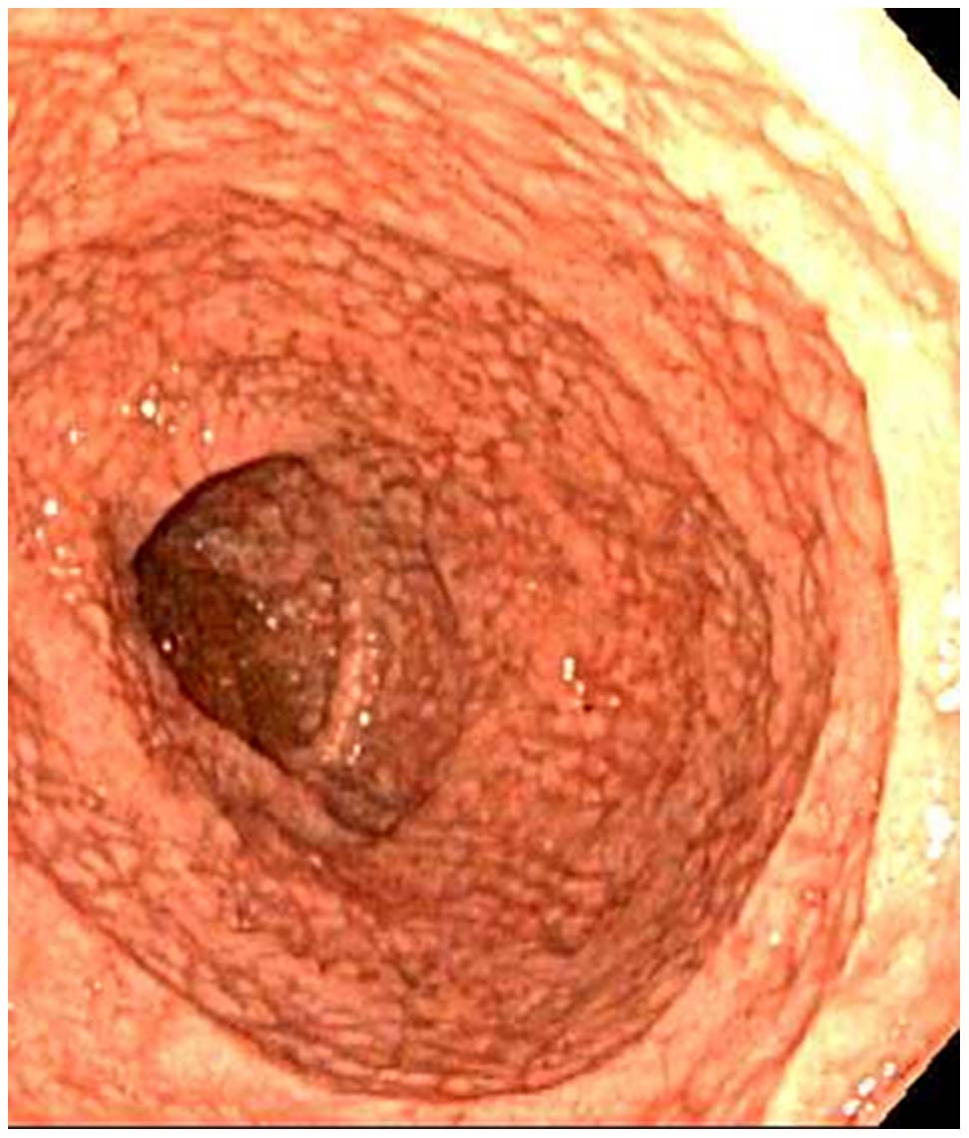
Duodenum as seen at endoscopy demonstrating nodular mucosa with scalloping of folds (Case 3).

We identified 3 cases of primary small intestinal CD4+ T-cell lymphomas, which occurred in 2 males and 1 female (ages 37, 50 and 53), all of western European descent. All patients had prior evaluations at other institutions for diarrhea and weight loss and were diagnosed as having celiac disease. They were referred to our institution for further work-up and management of celiac disease “non-responsive” to a gluten free diet (GFD). Pertinent clinical features of the patients are summarized in Table 1. On endoscopy, all patients had nodular mucosa with either flattening of folds or scalloping, manifesting a mosaic pattern (Fig. 1). Serologic tests for anti-gliadin and anti-tissue transglutaminase antibodies were negative on a gluten containing diet. None of the patients lived in areas endemic for HTLV-1 infection and serology for HTLV-1 was negative in one patient tested (Case 2). No consistent association with celiac disease-associated HLA-DQ alleles was seen. Two patients (Case 1 and 3) had mild elevations in LDH and beta-2 microglobulin at presentation. No other significant laboratory abnormalities were detected in any patient, with the exception of one patient (Case 1) who had persistent mild microcytic anemia (Hgb range 10-8-12.8). All had good performance status and low-risk IPI at diagnosis. PET-CT scans showed mild mesenteric lymphadenopathy with mildly increased FDG-avidity in one patient at diagnosis (Case 1). Liver lesions were seen on CT scan in one patient when he developed large cell transformation (Case 2), which led to multiple small bowel perforations and death due to sepsis 11 years post diagnosis. Two patients are alive with persistent lymphoma 4.6 and 2.5 years after diagnosis.

### Morphology

Multiple biopsies from the stomach, duodenum, jejunum, ileum, and colon were obtained at diagnosis and follow-up for all patients (Case 1, number of endoscopic procedures = 5, mean time interval between procedures = 0.8 years; Case 2, n = 11, mean time interval = 1 year; Case 3, n = 4, mean time interval = 0.75 years).

**Figure 2:**
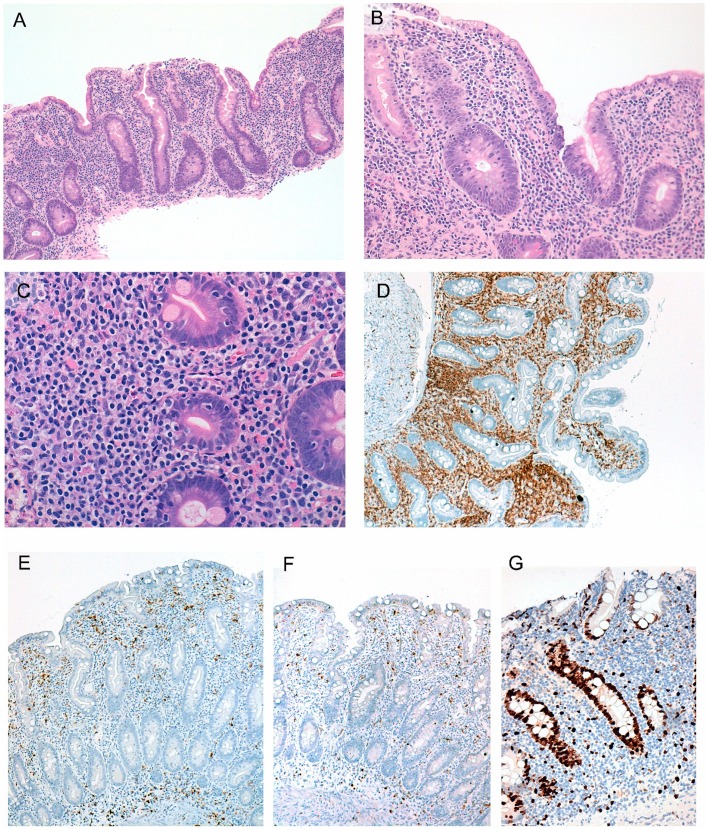
Representative photomicrographs of duodenal biopsies. Severe villous atrophy is seen (A) without any increase in intraepithelial lymphocytes (B). The lamina propria is expanded by a dense infiltrate of predominantly small-sized lymphocytes with evidence of crypt destruction and a lymphoepithelial lesion (C). On IHC analysis, the lymphocytes are CD4+ (D), CD5− (E), and CD7− (F), and have a low Ki-67 proliferation index (<5%)(G).

All small intestinal biopsies demonstrated crypt hyperplasia and variable degrees of villous atrophy, ranging from partial to total (Fig. 2a, Table 2). The most distinct feature was an expansion of the lamina propria by a patchy or dense sheet-like infiltrate of small to intermediate-sized lymphocytes (Fig. 2b). The lymphocytes had ovoid or irregular nuclei, fine chromatin, indistinct or small nucleoli, and scant or moderate clear cytoplasm (Fig. 2c). Only occasional large lymphocytes were noted. Mitoses were rare. No angiocentricity or angioinvasion was noted and no ulceration or necrosis was seen. Intraepithelial lymphocytes (IELs) were not increased (range 10–24/100 epithelial cells). However, duodenal biopsies from all patients showed foci of crypt destruction and distinct lymphoepithelial lesions (LELs) in the lower aspects of villi or crypts (Fig. 2c). Variable numbers of plasma cells, mostly in the superficial lamina propria, and eosinophils were present. Scattered, small lymphoid aggregates were seen. No granulomas were observed in any case.

**Figure 3:**
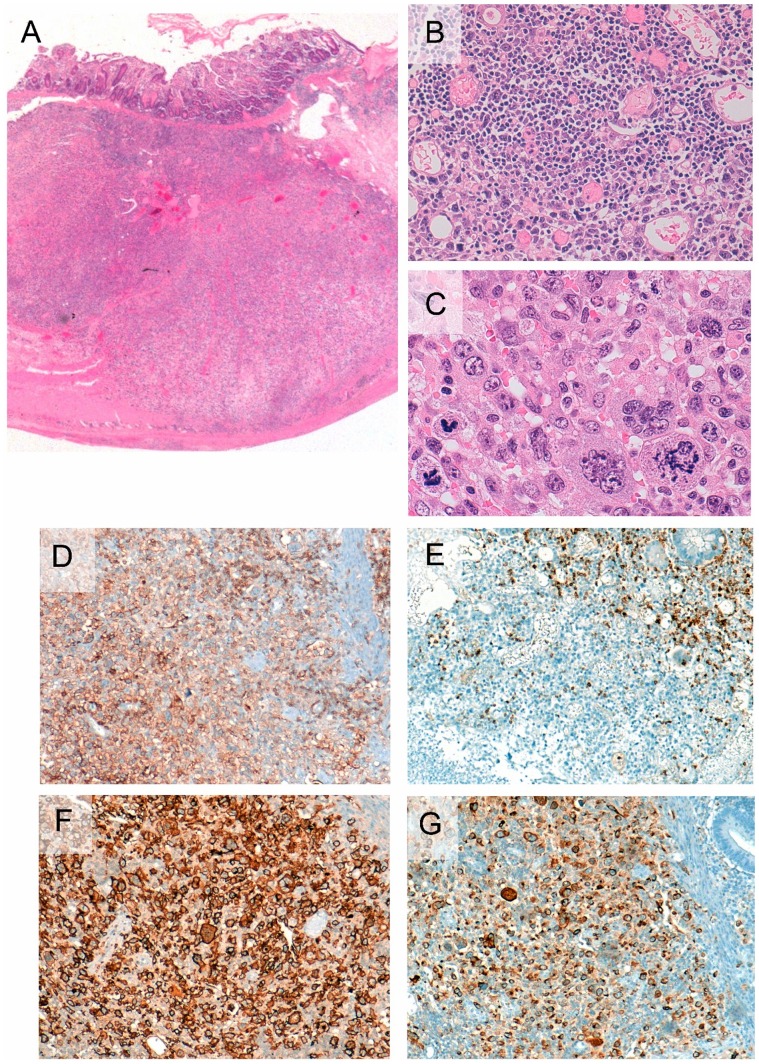
Section from a small intestinal resection specimen showing large cell transformation (Case 2). Transmural infiltrate of neoplastic lymphocytes is observed (A). The lamina propria shows admixtures of small and large atypical lymphocytes, including bizarre and multinucleated forms (B and C). The large lymphocytes express CD4 (D), CD30 (F) and granzyme B (G) and lack CD5 expression (E).

Sections of the proximal small intestinal resection specimen obtained at the time of exploratory laparotomy for jejunal perforation, 11 years post-diagnosis of the indolent lymphoma (Case 2, Table 2) showed persistence of small-sized lymphocytic infiltrate in the lamina propria, as well as crypt hyperplasia and total villous atrophy. In addition, an extensive infiltrate of large atypical lymphocytes was present, extending from the mucosa to the serosa (Fig. 3a–b). These lymphocytes exhibited marked nuclear pleomorphism, vesicular nuclei, prominent nucleoli, and abundant cytoplasm. Scattered bizarre, multinucleated lymphocytes, foci of necrosis, and frequent mitotic figures were identified (Fig. 3c).

**Figure 4:**
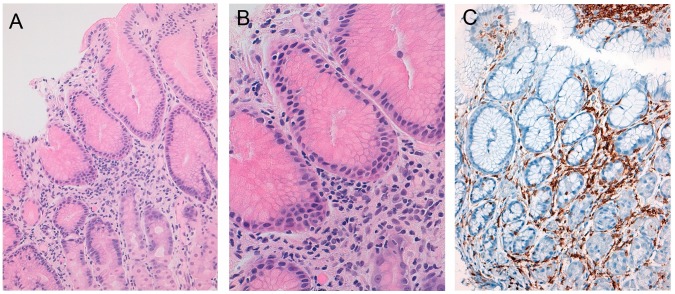
Representative photomicrograph of a gastric biopsy (Case 3). A mild lymphocytic infiltrate is present (A) with “rimming” of gastric glands (B). The lymphocytes express CD4 (C).

Mild lymphocytic infiltrates were seen in the gastric antral and fundic lamina propria (Case 2 and 3), occasionally rimming the gastric glands (Fig. 4) and in the lamina propria of the ascending colon in two patients (Case 2 and 3), which were almost imperceptible on morphologic evaluation, requiring IHC and molecular analysis for detection.

### Immunophenotype

The phenotypic profile of the neoplastic lymphocytes determined by IHC and flow cytometry is described in Table 2. A marked predominance of CD4+ T-cells was observed in all cases. These lymphocytes expressed CD3 (surface and cytoplasmic) and CD2. Downregulation or loss of CD5 or CD7 was seen in biopsies of 2 patients (Fig. 2d–f). The lymphocytes did not express BCL6, CD10, programmed death 1 (PD-1), Mum1/IRF4, FoxP3, CD8, CD25, CD30, CD56, TIA-1, perforin, granzyme-B or TCRγ. A mild infiltrate of cytotoxic CD8+ T-cells and B-cells was seen admixed. PD-1+ lymphocytes were seen as scattered cells in the lamina propria or forming loose clusters around lymphoid aggregates, overall accounting for <5% of all lymphocytes. We evaluated expression of Sirtuin (silent mating-type information regulation 2 homolog of Saccharomyces cerevisiae) 1 (SIRT1), a deacetylase, as a possible cause of FoxP3 non-expression/loss of expression by the neoplastic lymphocytes. However, only rare nuclear SIRT1+ lymphocytes were detected in two of the three cases. The IELs at the tips and along the lateral edges of the villi expressed CD8, while the LELs in the lower aspects of the villi and in the crypts were composed of CD4+ T-cells. The Ki-67 proliferation index was low (<5%) in all cases (Fig. 2g) and in situ hybridization for EBER was negative. Flow cytometry confirmed the phenotype of the neoplastic lymphocytes (CD4 expression in all cases) and also demonstrated expression of surface TCRαβ and lack of CD103 expression.

The neoplastic T-cells in the small intestine resection specimen from the patient exhibiting large cell transformation 11 years post-diagnosis and a couple of months prior to his demise (Case 2) showed additional phenotypic aberrations including expression of CD30, CD25, Mum1/IRF4, perforin and granzyme-B (Fig. 3). No ALK1 expression was seen.

No T-cell population displaying an aberrant phenotype was detected in the peripheral blood of any patient at diagnosis. However, an abnormal T-cell population was detected in the peripheral blood at the time of large cell transformation, which exhibited a phenotype similar to that seen in the small bowel (Case 2).

### T-cell Receptor Gene Arrangement Analysis

PCR analysis for TCRβ gene rearrangement, performed on biopsies from multiple sites in all patients, showed clonal products (Table 2). Identical sized products persisted in all subsequent samples from each patient, and the large cell transformation, which occurred in one patient (Case 2), appeared clonally related to the prior low grade intestinal lymphoma. The only exception was case 1 where polyclonal products were detected in the duodenal biopsy 15 years prior to diagnosis of lymphoma. Clonal products were only detected in the peripheral blood in one patient (Case 2), commencing four years after diagnosis and persisting till the patient’s demise, which were identical in size to those detected in the small intestine.

### SNP Array Analysis

All gains and losses were verified manually to determine erroneous calls and identify mosaic copy number changes (CNCs) undetected by the software. Analysis was restricted to CNCs >200 kb in length. Many of these CNCs occurred in regions of known variation found in normal individuals. For detection of pathogenic lesions, changes identified by array were compared with those in the Database of Genomic Variants (http://projects.tcag.ca/variation) and our own internal reference controls, to exclude known copy number variants (CNVs). Paired tumor and normal sample were analyzed for one patient (Case 3). This patient showed no pathogenic CNCs except subclonal loss of the X chromosome (monosomy X) seen only in the tumor sample. Analyses performed on multiple samples at different stages of disease from one patient (Case 2) detected diverse abnormalities, including loss on chromosome 19q in a sample obtained shortly after diagnosis and gains of 1p and 8q in a sample obtained 7 years later, while analysis of the premortem sample exhibiting large cell transformation showed multiple gains involving regions on chromosomes 1, 15, 17, and X and loss of heterozygosity on chromosome 7q (Table 2). A list of genes in the regions of CNCs was obtained from the UCSC Genome Browser [NCBI36/hg18 or GRCh37/hg19] (Table S1) and cancer associated genes were then manually curated and also compared with the Cancer Gene Census (COSMIC v61 Release; http://www.sanger.ac.uk/genetics/CGP/Census/). Candidate cancer associated genes are listed in Table 2.

## Discussion

In this report, we describe a series of three cases of a rare subtype of primary small intestinal CD4+ T-cell lymphoma associated with a relatively indolent disease course. To the best of our knowledge, a lymphoma with similar features was first reported by Carbonnel et al. in 1994, which was subsequently also published as part of a four case series. [14], [15] Thereafter, single case reports by Zivny et al. and Svrcek et al. also described what appear to be similar types of lymphomas (Table 3). [16], [17] The morphologic and immunophenotypic characteristics of our and previously published small intestinal CD4+ T-cell lymphomas are distinct from other primary T-cell lymphomas of the GI tract, such as EATL types I and II. The lymphocytic infiltrates in these lymphomas comprise a monotonous population of small to intermediate-sized cells that show mild pleomorphism and virtual restriction to the lamina propria. A generalized increase in IELs is usually lacking. Carbonnel et al. and Svrcek et al. reported an absence of LELs in their cases, however LELs were observed in all our cases and also in the case reported by Zivny et al.[14]–[17].

**Table 3:** Clinical and pathological characteristics of published cases.

Patient	Age/Sex	Symptoms/Signs	Small intestine histopathology	Immunophenotype	TCR	Sites of involvement	Therapy	Outcome
Carbonnel 1 [14]	43M	Diarrhea, weight loss	Small pleomorphic lymphocytes in LP, Normal villi to mild atrophy	CD2+, CD3+, CD5+, CD7+, **CD4+**, CD8−, CD57−	ND	Duodenum, jejunum, ileum, colon, liver	Chlorambucil, tetracycline, cyclophosphamide, teniposide, prednisone×10 cycles, holoxan, doxorubixin, etoposide×6 cycles.	Died of disease with intestinal obstruction, colon and liver involvement and ascites (14.6 years after diagnosis)
Carbonnel 2 [14], [15]	28M	Diarrhea, weight loss, small bowel volvulus	Small pleomorphic lymphocytes in LP, Normal villi to mild atrophy	CD2+, CD3+, CD5+, CD7+, **CD4+**, CD8−, TCRδ-, βF1+, **CD103**−**,** CD57−	Clonal	Duodenum, jejunum, liver, mediastinal lymph node, lung, skin, peripheral blood	Cyclophosphamide, doxorubicin, prednisone×4 cycles, Chlorambucil, 2-deoxycoformycin	Died of progressive multifocal leukoencephalopathy (4.8 years after diagnosis)
Carbonnel 3 [14]	59F	Diarrhea, weight loss, night sweats	Small pleomorphic lymphocytes in LP, Normal villi	CD2+, CD3+, CD5+, CD7+, **CD4+**, CD8−, TCRδ-, βF1+, **CD103**−	Clonal	Duodenum, jejunum, ileum	MACOP-B×3 cycles, cyclophosphamide, teniposide, prednisone×6 cycles, tetracycline.	Alive with disease (5.5 years after diagnosis)
Carbonnel 4 [14]	57M	Diarrhea, weight loss	Small pleomorphic lymphocytes in LP, total villous atrophy	CD2+, CD3+, CD5+, **CD7**−, **CD4+**, CD8−, TCRδ-, βF1+, **CD103**−**,** CD57−	Oligo-clonal	Stomach, duodenum, jejunum, ileum, isolated granulomas in liver	Chlorambucil and steroids.	Alive with disease (2.1 years after diagnosis)
Zivny [16]	60M	Diarrhea, weight loss	Blunt villi, lymphocytes in LP	CD3+, **CD4+**, CD8−, **CD103**−	Clonal	Stomach, duodenum	CVP×6 cycles.	Alive with disease (7 years after diagnosis)
Svrcek [17]	23M	Diarrhea, weight loss, intestinal obstruction	Small pleomorphic lymphocytes in LP, mild villous atrophy	CD2+, CD3+, CD5+, **CD7**−**, CD4+**, CD8−, βF1+, **CD103**−	Clonal	Duodenum, ileum, mesenteric lymph nodes, bone marrow	Prednisone, ACVB-P and anti-CD52	Alive with disease (3 years after diagnosis)

TCR: T cell receptor β or γ gene rearrangement; LP: lamina propria; ND: not done; MACOP-B: methotrexate, ARA-C, cyclophosphamide, oncovin, prednisone, bleomycin; CVP: cyclophosphamide, vincristine, prednisone; ACVB-P: doxorubicin, cyclophosphamide, vindesine, bleomycin, prednisone.

The neoplastic lymphocytes in EATLs can be small or intermediate in size, but they are of cytotoxic T-cell lineage as evidenced by cytotoxic granule protein expression, manifesting a double negative (CD4− CD8−) phenotype or displaying variable degrees of CD8 and CD56 expression. [7], [8] EATL type I shows frequent loss of surface T-cell receptor (TCR)αβ and nearly all cases express CD103, which is considered evidence of its origin from IELs, as this antigen is expressed by normal IELs. [7], [22] EATL type II comprises lymphomas of both the TCRαβ and TCRγδ lineages, with a subset of cases showing CD103 expression. [8], [23], [24] By contrast, all our lymphomas and all prior reported cases expressed CD4 and surface TCRαβ (or βF1) and all evaluated cases lacked CD103 and CD57 expression, consistent with a helper-inducer T-cell lineage. Of note, at the time of large cell transformation, which occurred in one of our patients, the neoplastic cells acquired CD30 and cytotoxic granule antigen expression and manifested cytomorphologic features indistinguishable from EATL type I or anaplastic large cell lymphoma.

The cell of origin of indolent small intestinal CD4+ T-cell lymphomas is not known at present. The phenotypic findings argue against an intraepithelial lymphocyte origin or derivation from natural or induced regulatory T-cells (Tregs). [25], [26] It is known that epigenetic regulation of the *FoxP3* gene, as well as post-translational modifications of the FoxP3 protein, modulate cellular FoxP3 levels and stability and hence Treg differentiation and function. [27] Recent studies have shown that acetylation of FoxP3 by the histone acetyltransferase p300 prevents polyubiquitination and its proteasomal degradation leading to increase protein stability and increased Treg suppressive capacity, while deacetylation by Sirtuin 1 (Sirt1), an NAD(+) dependant class III histone/protein deacetylase, leads to diminution or loss of FoxP3 protein and inhibition of Treg function. [28], [29] Thus, we investigated whether aberrant or deregulated expression of SIRT1 could explain the lack of FoxP3 expression by the neoplastic CD4+ T-cells. Using a specific monoclonal antibody, we found no convincing evidence of this phenomenon in any of our cases. The morphologic, phenotypic, and clinical features of indolent small intestinal CD4+ T-cell lymphomas bear some resemblance to primary cutaneous CD4+ small/medium sized pleomorphic T-cell lymphomas, which are thought to be of T-follicular helper (T_FH_) cell origin. [30] No expression of CD10, BCL6 or PD-1 was observed in any of our cases. However, given the diversity and plasticity of T_FH_ cells, evaluation of additional T_FH_ markers needs to be investigated in future studies to explore the possible origin of indolent small intestinal CD4+ T-cell lymphomas from a particular T_FH_ subtype, or another functional subset or lineage of CD4+ T-cells. [31].

Presence of phenotypically aberrant lymphocytes and clonal TCRβ gene rearrangements in the stomach and colon in two of our cases indicated relatively early GI disease dissemination, which is also a common occurrence in refractory celiac disease (RCD) type II (a clonal indolent precursor of EATL type I) and EATL type I, having been reported in up to 78% and 54% of cases, respectively. [32], [33] Zivny et al. and Carbonnel et al. documented cases with neoplastic T-cell infiltration of the stomach, but colonic involvement was not described in any of the prior reports (Table 3). [14], [16] In contrast to the frequent extra-GI dissemination at presentation in RCD type II or EATL type I, detected in 44% and 43% of patients, respectively,[7], none of our patients and previously published cases had evidence of systemic disease at presentation. However, systemic dissemination does occur later in disease course. Currently, there are no guidelines regarding the use of a particular modality to detect and monitor low level extra-GI disease dissemination in individuals with indolent small intestinal CD4+ T-cell lymphomas and data regarding systematic peripheral blood analysis by flow cytometry and PCR for TCRβ rearrangement are lacking. Hence, the time of occurrence and frequency of peripheral blood involvement is not known at present. We detected a circulating T-cell clone in the peripheral blood in only one patient, in the absence of systemic disease, four years post-diagnosis, by PCR analysis, but not by flow cytometry, highlighting the utility of the former modality in disease monitoring.

The relationship of the described indolent small intestinal CD4+ T-cell lymphomas with other rare types of indolent GI lymphoproliferative disorders (LPD) published in the English literature is unclear. The clonal CD4+ T-cell LPDs involving the GI tract reported by Hirakawa et al. and Egawa et al. showed similar cytomorphologic features as our and other aforementioned published cases, but differed with regard to their clinical presentation and endoscopic features or phenotype. [13], [18] Hirakawa et al. detected multiple polypoid lesions in the stomach, duodenal bulb and terminal ileum, on endoscopic and radiologic imaging, in an asymptomatic 47 year old male, which were covered by normal appearing mucosa. [18] The jejunum was spared and the colonic mucosa showed apthous ulcers. The neoplastic lymphocytes at all sampled locations expressed CD45RO, CD2, CD3, CD4 and CD103(HML-1) and the lymphoma persisted one year after chemotherapy (cyclophosphamide, vindesine, pirarubicin and prednisolone×4) and approximately two years post-diagnosis. Egawa et al. described a low grade CD3+, CD4+/−, CD56−, CD57− and CD103(HML-1)- T-cell LPD in a 51 year old male, manifesting as multiple, clonally related, oral and colonic ulcers that had a punched out appearance and displayed a waxing and waning course over 17 years, with sparing of the esophagus, stomach and duodenum. [13] The ulcers were refractory to steroid and antiviral (acyclovir) therapy and although spontaneous healing of the oral ulcers was seen after two years of follow-up, the colonic ulcers persisted. Ranheim et al. also reported an indolent LPD in a 35-year old male that had a predilection for the oral and colonic mucosa, presenting as clonally related and recurrent, self-healing ulcerating lesions over a 9 year period. [12] However, in contrast to the case of Egawa et al., the neoplastic lymphocytes expressed CD8. A TCRαβ lineage was established and a lack CD56 and TIA-1 expression was reported. The recently described indolent NK-cell LPDs can involve the small intestine but the neoplastic cells characteristically express CD56 and lack CD4 expression. [19] Distinguishing indolent small intestinal CD4+ T-cell lymphoma from RCD type II is facilitated by the lack of significant intraepithelial lymphocytosis and predominant lamina propria involvement in the former, as well as differences in the immunophenotype. [35].

Discriminating between indolent small intestinal CD4+ T-cell lymphoma and secondary GI involvement by certain subtypes of PTCL, especially angioimmunoblastic T-cell lymphoma, HTLV1-associated adult T-cell leukemia/lymphoma, mycosis fungoides and T-cell prolymphocytic leukemia/lymphoma, can be challenging on morphologic and phenotypic grounds. [36]–[41] The presentation of secondary lymphomatous involvement of the intestine is generally more acute and there is usually evidence of disease elsewhere. HTLV-1 serology was negative in one of our patients tested and in all prior cases where evaluated. [14] As mentioned above, although all our cases and previously published cases lacked clinical evidence of systemic disease at presentation, dissemination to other organs, including liver, skin and lungs, which occurred later in disease course, could pose difficulties in ascertaining the primary site of these lymphomas if the patient presents with long standing disease. [14].

Differentiating inflammatory disorders, especially celiac disease, inflammatory bowel disease or autoimmune enteropathy from indolent small intestinal CD4+ T-cell lymphomas can be diagnostically challenging as the mucosal architectural alterations can be similar, the neoplastic lymphocytic infiltrate can be patchy at times and aberrations of the T-cell antigens are absent in some cases (Table 3). [42], [43] Moreover, eosinophilia and epithelioid granulomas have been reported in the small intestine and other organs of individuals with these lymphomas, at times in the absence of significant lymphocytic infiltration. [14], [17] Hence, it is not hard to imagine why all of our and many of the published cases were misdiagnosed as celiac disease or an inflammatory disorder, leading to a considerable delay in treatment in many instances. Phenotypic, molecular and genetic analyses are helpful, but a high index of clinical suspicion is essential for diagnosing this subtype of lymphoma.

The demographic features of our patients are similar to those described for individuals with EATL type I [7] and distinct from individuals with EATL type II, who tend to be older (7^th^ decade) and are of diverse ethnicity/race. [8], [24] It should be noted that indolent small intestinal CD4+ T-cell lymphomas can occur in young adults. [14], [17] Patients with such lymphomas can manifest similar clinical signs as those with EATL types I and II (diarrhea and weight loss), but the latter more often have an acute presentation with intestinal obstruction or perforation. [7], [8] The incidence of indolent small intestinal CD4+ T-cell lymphomas is not known at present. Given the scarcity of reported cases they appear quite rare. However, given the overlapping morphologic features with other types of intestinal lymphomas and the clinical presentation that mimics an inflammatory disorder, we believe that indolent small intestinal CD4+ T-cell lymphomas are currently under-recognized, especially in limited resource settings. A recent Asian study of EATL type II reported two cases of CD4+ T-cell lymphomas, as part of a large series, however specific pathologic and clinical characteristics of these cases were not provided. [23] A rising incidence of EATL has also been reported in the US and it remains to be seen whether inclusion of non-EATL type I cases such as indolent small intestinal CD4+ T-cell lymphomas are contributing to this increase. [44].

Our results and the published reports indicate low morbidity and long survival of patients with indolent small intestinal CD4+ T-cell lymphomas, often exceeding a decade [14], [16], [17] in contrast to the median overall survival of 10 and 7 months, respectively, for patients with EATL types I and II. [7], [23] Optimal management of patients with indolent small intestinal CD4+ T-cell lymphomas is unclear. Therapy with oral or luminal, non-absorbable steroids led to good symptom control in our patients, although none achieved remission, similar to prior reports. [14], [16], [17] Aggressive therapy with a variety of chemotherapy regimens in the past has also been unsuccessful (Table 3). Newer therapeutic modalities are needed due to the potential for disease progression, transformation and death.

The etiology of indolent small intestinal CD4+ T-cell lymphomas is unclear. Persistent antigenic stimulation as a consequence of an immune-mediated or infectious disorder is a possibility. One of our patients demonstrated a polyclonal T-cell infiltrate in conjunction with villous atrophy 15 years before diagnosis, suggesting evolution from an antecedent inflammatory disorder. Due to a lack of material available for evaluation during the interim period, the precise time of neoplastic transformation in this patient is unknown. Results of TCRβ variable region gene spectratyping and high-throughput, next generation sequencing are awaited to investigate the presence of skewed T-cell repertoires with preferential usage of certain TCRβ gene family members or overrepresentation of certain motifs in the complimentary determining regions, which could suggest a role of autoantigens or infectious agents in disease pathogenesis. No association with celiac-disease associated HLA alleles has been discerned for indolent small intestinal CD4+ T-cell lymphomas [14], but the presence of other genetic predisposing factors cannot be ruled out. These lymphomas lack evidence of EBV infection by in situ hybridization or serologic assays when analyzed. Only one case in the series of Carbonnel et al. showed increased EBER+ cells, which could reflect an expansion of latent EBV infected B-cells in the setting of lymphoma associated immune dysregulation. [14].

Prior to this report, systematic genomic evaluation of indolent small intestinal CD4+ T-cell lymphomas was lacking. One case each, reported previously, displayed trisomy 5 and t(4;16)(q26;p13.1) by conventional cytogenetic analysis. [14] Cloning and characterization of the breakpoints of the t(4;16)(q26;p13.1) led to the identification of the B-cell maturation antigen (BCMA) gene that was fused to the interleukin (IL)2 gene. [45] BCMA is a member of the tumor necrosis factor receptor superfamily, which plays critical roles in B-cell survival and proliferation, but is not expressed to any significant degree in T-cells. [46] The pathogenic significance of the chimeric transcript or the gain or loss of function of either partner gene as a consequence of the translocation is not known.

Our analysis, using SNP-arrays, indicates relative genomic stability during the early phases of disease and a lack of recurrent genetic abnormalities. Aberrations associated with EATL types I and II, such as amplifications of 9q31.3, deletions of 16q12.1 or 1q and 5q amplifications, were not detected, with the exception of an 8q gain observed in one sample. [47]–[49] Interestingly, sequential analysis of samples from one patient revealed different abnormalities at different time points, suggesting either the presence of an oligoclonal proliferation, not detectable by PCR analysis, which underwent therapy-related clonal selection or the occurrence of distinct cytogenetic abnormalities over time in an otherwise clonal T-cell lymphoproliferation. Complex cytogenetic abnormalities observed on disease transformation included gains involving loci harboring potential oncogenes (Table 2). Of these, *STAT3* is a known oncogene that is deregulated in different types of T-cell lymphomas, including anaplastic large cell lymphomas and cutaneous T-cell lymphomas, [50] while *ZFX*, a gene important for stem cell renewal, though not implicated in T-cell malignancies, has been shown to have increased expression in solid tumors. [51]–[53].

In summary, we report a series of unique primary small intestinal CD4+ T-cell lymphomas exhibiting an indolent clinical course that are frequently misdiagnosed as celiac disease. Our findings, in conjunction with prior reports, suggest that these lymphomas represent a distinct entity. A comprehensive multimodality approach helps in diagnosis. Optimal management is presently unclear, but close follow-up is recommended as disease progression and transformation does occur. Greater awareness of this entity among clinicians and pathologists should lead to the discovery of more cases, which could facilitate elucidation of the etiology of these lymphomas and development of targeted therapy.

## Supporting Information

Table S1 Details of copy number changes detected by SNP array analysis.
(XLS)
